# The dietary fiber and micronutrient composition of traditional foods from Lebanon and their contribution to dietary adequacy: A call for action

**DOI:** 10.1371/journal.pone.0312429

**Published:** 2024-10-29

**Authors:** Maha Hoteit, Edwina Zoghbi, Alissar Rady, Iman Shankiti, Yonna Sacre, Lara Hanna-Wakim, Rana Mahfouz, Carla Ibrahim, Ayoub Al-Jawaldeh

**Affiliations:** 1 Faculty of Public Health, Section 1, Lebanese University, Beirut, Lebanon; 2 Food Sciences Unit, National Council for Scientific Research Lebanon (CNRS-L), Beirut, Lebanon; 3 Country Office for Lebanon, World Health Organization, Beirut, Lebanon; 4 Department of Nutrition and Food Sciences, Faculty of Arts and Sciences, Holy Spirit University of Kaslik (USEK), Jounieh, Lebanon; 5 Department of Agricultural and Food Engineering, School of Engineering, Holy Spirit University of Kaslik (USEK), Jounieh, Lebanon; 6 World Health Organization Regional Office for the Eastern Mediterranean, Cairo, Egypt; University of Ha’il, SAUDI ARABIA

## Abstract

Traditional Lebanese cuisine is based on traditional dishes, where Arabic sweets play an important role in daily consumption. This study focuses on the evaluation of total fibers and trace elements, especially vitamins A, D, E, and C of traditional foods and Arabic sweets commonly consumed in Lebanon by chemical analysis. A total of thirty types of Arabic sweets were chosen from reputable confectionery establishments, while thirty varieties of traditional Lebanese dishes were collected from central kitchens in the main Lebanese governorates. It was discovered that 23% percent of Arabic sweets and 30% of traditional dishes were rich in total dietary fiber. Moreover, Arabic sweets had trace amounts of vitamin A, vitamin E, and vitamin C. In specific sweets, vitamin A content showed variability, with values ranging from 8ug to 15 ug per 100 g of edible portions. Most of the traditional dishes contained traces of vitamin C. However, Tabboula stood out as the only dish that contributed to over 23% of the recommended daily value for vitamin C. Trace amounts of vitamins A, D, E, and C were present in almost all traditional Lebanese foods and Arabic sweets. This study revealed that these foods lack essential micronutrients and total dietary fibers.

## Introduction

Globally, people’s eating habits are drastically changing, prioritizing convenience over the nutritional value of traditional foods [1]. In Lebanon, known for its diverse culinary heritage, traditional foods are essential for preserving cultural identity and fostering social unity. Traditional foods are defined as those consumed within a specific community or region for an extended period, distinguished by their historical significance, local ingredients, and traditional preparation methods. Whether produced at home or bought from neighborhood stores, traditional meals and Arabic sweets continue to play a significant role in people’s daily diets [2]. It is emphasized that studying the total fibers and micronutrient content of these foods is vital for enhancing public health and overall wellness. Lebanese cuisine boasts a wide range of micronutrients that are essential for maintaining good health. Foods, such as carrots, leafy greens, citrus fruits, peppers, dairy products, fatty fish, nuts, and seeds, are packed with important vitamins like A, C, D, and E. These vitamins are crucial for supporting various bodily functions, including vision, immune health, collagen synthesis, bone health, and antioxidant defense [3]. Additionally, the abundance of dietary fibers found in fruits, vegetables, legumes, and whole grains in Lebanese dishes plays a vital role in promoting digestive health, satiety, and blood sugar regulation. Dietary fibers support the health of the gut microbiota and the corresponding host health [4]. The Mediterranean diet (Med-diet) is a plant-based diet that shares a lot of similarities with the Lebanese traditional diet as it includes high amounts of olive oil, fresh vegetables, fruits, grains, chickpeas, lentils, bulgur wheat, fish, and a variety of herbs and spices whereas moderate amounts of dairy, poultry, and fish, with red meat that are consumed less frequently. Consequently, traditional dishes encompassing the med-diet play an important role in defining the Mediterranean Diet and its preventive advantages for cardiometabolic health [5]. Furthermore, the Mediterranean diet has been proven to have a preventive impact on cardiovascular diseases and cognitive impairment, not only in Mediterranean populations but also in non-Mediterranean populations [6]. In 2020, 88.3% of the population in Low and Middle-income countries (LMCIs), including Mediterranean countries, struggled to afford a nutritious diet. Moreover, two billion suffered from micronutrient deficiencies [7]. Hidden hunger, unlike protein-energy malnutrition, refers to the covered health impact of consuming an energy-dense but nutrient-poor diet, leading to various micronutrient deficiencies, which can occur independently of decreased energy intake [8]. More than two billion people are thought to be affected globally, especially in low- and middle-income nations where there is a reliance on inexpensive food staples and little variety in the diet [9]. Approaching the midpoint of the United Nations Decade for Action on Nutrition, it is fitting to evaluate the strategies employed to combat hidden hunger and the progress made towards achieving Sustainable Development Goal number two, which aims to eradicate hunger, ensure food security and enhance nutrition, and promote sustainable agriculture [10]. The first step in this roadmap starts by filling the gap in published data regarding the nutritional characteristics of traditional dishes including total fiber and micronutrient’s content. A research team has recently started shedding light on the nutritional value of traditional foods and recipes at a national level by conducting a study in Lebanon. This initiative aims to assess the dietary intake of the population and to provide dietary information, preserve culinary heritage, and reduce the risk of cardiometabolic diseases [11–14]. Consequently, a database analysis was created to examine the fatty acid profile and macronutrient makeup of common Lebanese recipes. Results showed that while most of the recipes had modest sugar levels, more than 60% of the samples had excessive salt content. Moreover, more than 80% of the samples had poor amounts of iron in all Lebanese governorates [13]. In addition, in 75% of these food items, there was a lack of unsaturated fatty acids. In terms of saturated fatty acids, the ratio of polyunsaturated to monounsaturated (P.M.S) was below the recommended 1:1:1 ratio in 96% of the samples [14]. Like many other countries, Lebanon has witnessed shifts in dietary habits, from traditional diets rich in fruits, vegetables, and whole grains to more Westernized diets high in processed foods, sugars, and unhealthy fats. This transition has contributed to an increase in obesity rates and diet-related chronic diseases [15]. Concurrently, various studies on the dietary habits of adults show a nutrition transition in the population, marked by higher consumption of total fats, Trans fatty acids, and salt [16]. Over the last decade, the incidence of overweight and obesity has risen significantly, with 38% of adults being classified as overweight and 27% as obese [17]. Additionally, Lebanon’s current levels of hunger, malnutrition, and micronutrient deficiencies can be attributed to various factors such as political instability, climate change, and economic challenges exacerbated by the COVID-19 pandemic. Findings showed that during the pandemic, 9 out of 16 Lebanese households consumed less than 2 meals daily, with 70% skipped meals. Despite low food consumption, 82.4% used livelihood coping strategies, and most households experienced income and debt decreases to afford food. Dietary diversity was poor where a decrease in fruits, vegetables, meats, poultry, fish and dairy products was observed [18, 19]. Research on the nutritional status in Lebanon highlights a worrying increase in overnutrition, reflected by the high rates of non-communicable diseases [20]. Among Lebanese adults, the adherence to the Mediterranean diet scale was relatively low, with a score of 4.2. Although participants did meet the guidelines for consuming fruits, vegetables, dairy, legumes, and nuts, their intake of whole grains, poultry, and fish was below the recommended levels. Conversely, there was a higher consumption of meat observed [21].

Insufficient fiber intake is a prevalent issue among individuals aged 6 to 60, as their consumption falls below 50% of the recommended dietary allowances (RDA). Children between the ages of 4 and 13 often experience deficiencies in vitamins D (99%) and A (69.5%) [22]. Additionally, many preschoolers do not consume enough folate, calcium, and vitamin A, with a significant majority failing to meet their vitamin D requirements (91.9%) [23]. Furthermore, over half of individuals aged 6 to 20 consume less than two-thirds of the RDA for vitamin A, and a quarter of them are deficient in vitamin C. Similarly, adults aged 20 to 60 and older adults (>60 years) also struggle to meet two-thirds of the RDA for vitamins A and C [24]. According to the Global Fortification Data Exchange (GFDE), Lebanon lacks fortification of essential food staples such as maize flour, oil, and rice, except for salt, which has been fortified since 2008 as per a mandatory national law [25]. The GFDE program recommends fortifying food with micronutrients to improve dietary quality and address hidden hunger (Lebanon Fortification Dashboard, 2008 [25]. In the Middle East, commonly consumed food vehicles for fortification are cooking oil and flour, which are considered staple foods, while less commonly used options include orange juice and dairy products [26, 27]. The WHO and the Ministry of Public Health (MOPH) have prioritized food fortifications in their national nutrition strategy (2023–2025) to address the deficiencies in fibers and micronutrients in Lebanon, with the aim of incorporating these actions into national policies and guidance [28]. Thus, identifying the content of the frequently consumed traditional foods is critical to understanding the Lebanese traditional food status in micronutrients, total dietary fibers and their contributions for dietary adequacy that are needed to developing intervention strategies. The purpose of this study is to evaluate the micronutrient content and percentage of contribution in a 2000 kcal diet of 30 main Lebanese traditional dishes and 37 Arabic sweets by establishing a database analysis of vitamins A, D, E, C and total fibers.

## Materials and methods

### Food selection

This study aims to investigate the process of food sampling, with a specific focus on Arabic desserts and composite foods that are popular among the Lebanese community. The initial selection of these food items was based on their popularity and the frequency at which they are consumed [11–13]. Traditional composite dishes are meals that are typically consumed during main meals, such as lunch or dinner, and are made up of ingredients from at least three of the five main food groups. These dishes require culinary skills for their preparation [29]. A total of 30 commonly consumed traditional dishes were recognized and included in the analysis. The names of these dishes were noted based on the most used name at a national level, considering the ingredients used. The analysis focused on a total of 37 types of Arabic sweets. These specific sweets were selected as the most popular and commonly consumed products in the market.

### Food sampling

Batch 1 samples were gathered from a list of traditional foods. The list included: *Baba ghanouj*, *Batata mahchi*, *Borgul bi banadoura*, *Chichbarak*, *Falafel*, *Fatayer sabanikh*, *Fattat Hommos*, *Fattoush*, *Foul moudamas*, *Hindbe bil zet*, *Hommos bi tahini*, *Kafta wa batata*, *Kebba bil sayniya*, *Koussa mahchi*, *Lahm bil ajin*, *Loubia bil zet*, *Malfouf mahchi Moujadara*, *Moghrabia*, *Mousaka batinjan*, *Riz a dajaj*, *Riz bi lahma*, *Sayadia*, *Shawarma dajaj Shawarma lahma*, *Tabboula*, *Warak enab*, *Yakhnat Bamia*, *Yakhnat Fassoulia and Yakhnat Mouloukhia Mouloukhia*. To ensure the representation of regional food variations, 500 g of each of the 30 identified traditional foods (totaling 150 samples) were collected from five central kitchens situated in different Lebanese governorates: Tripoli, Beqaa, Beirut, Mount Lebanon, and South Lebanon. The selection of these central kitchens was random, considering factors such as their expertise in homemade dishes, local popularity, and their contribution to social entrepreneurship and women’s empowerment. In a similar fashion, batch 2 was comprised of samples of Arabic sweets. A total of 37 distinct types of Arabic sweets were gathered, with 500 g of each type collected from two different pastry shops that had different price ranges. The selected Arabic sweets were: *Baklava mixed*, *Baklava mixed light*, *Barazik*, *Boundoukia*, *Daoukia*, *Foustoukia*, *Ghourayba*, *Halawa*, *Halawa light*, *Halawat El jiben*, *Ish el bulbul*, *Kallaj kashta*, *Karabij joz maa crema*, *Katayef Kashta*, *Kounafa kashta bil kaak*, *Kounafa bil jiben*, *Maakaron*, *Maakroun wa moushabak*, *Maamoul tamer*, *Maamoul mad kashta*, *Maamoul mad joz*, *Maamoul fostok*, *Maamoul joz*, *Madlouka*, *Mafrouka kashta*, *Mafrouka fostok*, *Moufattaka*, *Mouhallabiya*, *Moushabak*, *Nammoura*, *Osmaliya*, *Riz bil halib*, *Saniora*, *Sfouf*, *Shaaybiyat*, *Ward el sham and Znoud El sitt*. This accumulation resulted in a total of 51 samples. The samples were then stratified based on the type of retail sale point, classifying them as either inexpensive or expensive. Sampling Arabic desserts was somewhat convenient; if they were sold at the pastry shops, they were obtained. In batch 2, the analysis involved studying the total fiber (soluble and insoluble), vitamins A, E, C, and D, as well as the nutritional elements that were previously examined in batch 1 traditional dishes. These elements included total fat, saturated fat, monounsaturated fat, polyunsaturated fat, trans fatty acids, salt, total sugar, vitamin D, iron, carbohydrates, ash, protein, and energy content. Details about the composition of both traditional dishes and Arabic sweets have already been published.

### Handling of food samples

The samples were handled, packaged, and transported in accordance with established protocols to prevent any modifications that could influence analytical measurements. The plans for moving primary samples from the collection locations to the laboratory were outlined in the transportation and storage protocol. The condition in which the samples were taken had to match the standard by which the producers offer them. The analysts were consulted during the specification of the storage process, which included the choice of containers and modes of transportation. During transit, secure storage in inert containers that could be heat-sealed using basic tools was necessary. The samples must be sent to the lab as soon as possible after being chilled with crushed ice or solid CO_2_. For certain governorates, driving a car or a motorcycle was an appropriate mode of transportation due to the small distance to the laboratory. On the other hand, cooled vehicles were needed for transportation across larger distances. Before the analysis started, three sample replicas were kept in the lab for practical and logistical reasons. The samples were kept frozen since, according to current common practice, that is typically the lowest permissible state and that –40°C or even –70°C is preferred. It was permissible to store samples at –20°C or –30°C for fat content analyses. The sample receptacles had minimal headroom and were well sealed.

### Chemical analysis

Five hundred grams of each food sample was mashed and examined in the lab. For further analysis, the remaining samples were kept frozen at -18°C in tight containers. The vitamins profile was assessed using different analytical methods under the supervision of the technical committee at the Industrial Research Institute Laboratories and following defined protocols based on the Association of Official Analytical Chemists (AOAC) [30]. (Check S4 Table for more details).

#### Vitamin C

The reagents were prepared by dissolving 15 g of glacial HPO_3_ in 40 ml of glacial CH_3_COOH and 150 ml of H_2_O. The resulting mixture was then diluted to 250 ml and filtered into a 500 ml beaker. In a separate container, 0.9 g of EDTA was dissolved in 200 ml of H2O to make a 250 ml solution. Just before use, equal amounts of the EDTA and HPO3 solutions were combined. The 1 mg/ml ascorbic acid standard solution was created by diluting 50 mg of ascorbic acid in a 50 ml volumetric flask with precipitant solution. For the indophenol standard solution, 0.0625 g of 2, 6-dichloroindophenol Na salt and 0.0525 g of NaHCO_3_ were dissolved in 50 ml of H_2_O, then diluted to 250 ml, filtered, and stored in the refrigerator. Three Erlenmeyer flasks with a capacity of 50 ml were utilized. Each flask was initially filled with 5 ml of a precipitant solution. Next, three separate 2.0 ml aliquots of ascorbic acid solution were added to the respective flasks. The flasks were then subjected to titration using an indophenol standard solution until a faint rose-pink color remained visible for a period of five seconds. Finally, three blank samples were titrated using 7 ml of the precipitant solution and 15 ml of H_2_O.


$$Ascorbic\;acid\;equivalent\;to\;1\;ml\;indophenol\;standard\;solution=\frac{2\;mg\;ascorbic\;acid}{ml\;dye‐\;\mathrm{blank}\;\mathrm{titration}}$$

Given that the blank titration is on average 0.1 ml, the equation can be further specified as:


$$Ascorbic\;acid\;equivalent\;to\;1\;ml\;indophenol\;standard\;solution=\frac{2\;mg\;ascorbic\;acid}{ml\;dye‐\;0.1\;ml}$$

The assay solution was prepared by mixing 5 g of a sample with 50 ml of precipitant solution in a 125 ml beaker, followed by stirring and filtering. This solution was then divided into three aliquots, each placed in separate 50 ml Erlenmeyer flasks, with aliquot volumes ranging from 5 to 20 ml depending on the anticipated vitamin C levels. The ascorbic acid content was measured by titrating these aliquots with an indophenol reference solution, while two blanks were similarly titrated without the sample. The titration process involved adding an equal volume of water to the test solution to achieve the color change endpoint [31]. The ascorbic acid concentration was calculated using the formula:

Where VS = Average ml for assay solution titration,

VB = Average ml for blank titration,

F = mg ascorbic acid equivalent to 1.0 ml indophenol standard solution,

V = volume of assay solution,

E = volume of aliqot,

WS = weight of sample.

#### Vitamin A

Homogenized samples ranging from 2 to 10 g were combined with 1 g of ascorbic acid, 150 ml of ethanol, and 50 ml of a 60% KOH solution in an Erlenmeyer flask. The flask was then covered and stirred overnight in the absence of light to facilitate saponification. Subsequently, the mixture was transferred to a separatory funnel and diluted with 150 milliliters of H_2_O. It was then subjected to three extractions using hexane as the organic solvent. The organic solvent was subsequently washed with water, dried using Na_2_SO_4_, and evaporated utilizing a rotary evaporator. The resulting residue was dissolved in a solution consisting of 2% isopropanol in hexane. For chromatographic analysis, a Lichosorb Si 60 column with specific conditions was used, and the sample was introduced into an HPLC system. To determine the concentration of the sample, a calibration curve was established by integrating the peak with a similar retention time as the standard [31].

#### Vitamin D

A mixture of homogenized samples weighing between 10 and 30 g was combined with 1 g of pyrogallol, 100 ml of ethanol, and 50 ml of a 50% KOH solution. The saponification step was carried out either under nitrogen for a duration of 20 to 45 minutes at a temperature ranging from 70 to 100°C, or by stirring the mixture overnight at room temperature in the absence of light and wrapped in aluminum foil. Once the saponification was complete, the resulting solution was transferred into a separatory funnel and mixed with 150 ml of H_2_O. This mixture was then subjected to three extractions using 100 ml of hexane each time. The organic solvent was separated and washed three times with 100 ml of H_2_O. It was then filtered over Na_2_SO_4_ and evaporated. To prevent oxidation, butylhydroxytoluene was added, and the remaining residue was dissolved in methanol. For chromatographic analysis, a C18 column was used with methanol as the mobile phase and UV detection at 265 nm. To construct a calibration curve, calibration solutions were injected, and sample solutions were tested accordingly. The concentrations of the samples were calculated from the curve based on the integration of peak [31].

#### Vitamin E

A standardized sample (ranging from 2 to 10 g) was mixed with 1 g of ascorbic acid, 150 ml of ethanol, and 50 ml of 60% KOH in an Erlenmeyer flask. The flask was then covered, wrapped in foil, and left to stir overnight in the dark for saponification. The resulting mixture was subsequently transferred to a separatory funnel, combined with 150 ml of H_2_O, and subjected to three extractions with 100 ml of hexane. The organic solvent obtained was washed three times with 100 ml of H_2_O, filtered through Na_2_SO_4_, and evaporated using a rotary evaporator. The remaining residue was dissolved in a mobile phase consisting of 3% dioxane in hexane. The HPLC analysis was carried out utilizing a Lichosorb Si 60 column with UV detection at 292 nm, a flow rate of 1.0 ml/min, and an injection volume of 20 μL. A calibration curve was established, and the sample concentration was determined based on the linearity of the curve and the retention time matching the standard [31].

#### Fibers

The total dietary fiber content was measured using the AOAC Method 991.43 [30], which involved the use of protease (Megazyme E-BSPRT), amyloglucosidase (Megazyme E-AMGDF), and thermostable-amylase (Megazyme E-BLAAM). A 50 mM buffer solution of 2-(N-morpholino) ethanesulfonic acid (MES) and tris(hydroxymethyl) aminomethane (TRIS) at pH 6.3 was prepared by dissolving 9.76 g of MES and 6.055 g of TRIS separately in water to make 1 L solutions. Furthermore, a stabilizing buffer was created with 50 mM MES–TRIS, 3 mM Ca^2^⁺ ions, and 10 mM Na⁺ ions by dissolving MES, TRIS, NaCl, and CaCl₂ in water to make 1 L solutions, all adjusted to pH 6.3 at 24°C. The instrumentation used in the study adhered to the guidelines outlined in AOAC Method 991.43 [31, 32].

### Percentage daily contributions in 2000 Kcal-diet

The United States Department of Agriculture (USDA) has provided guidelines for adults aged 19 years and above regarding their recommended daily values (DV). These guidelines suggest consuming 28 g of fiber, 900 micrograms (μg) of vitamin A, 15 μg of vitamin E, 90 mg of vitamin C, and 20 μg of vitamin D per day [33, 34]. Moreover, the Food and Drug Administration (FDA) stipulates that if a food item provides at least 20% of the daily value per reference amount, it qualifies as "high," "rich in," or an "excellent source of" nutrients. For foods supplying 10–19% of the DV for nutrients, they are labeled as a "good source," "contains," or "provides" the nutrient. Conversely, foods containing less than 10% of the DV for each nutrient per reference amount are considered to have low quantities and change all others [35]. The determination of daily values for total fiber, vitamin A, vitamin D, vitamin E, and vitamin C was based on selected dietary standards.

## Results and discussion

### Dietary fibers and micronutrients profile

#### Dietary fibers

Our examination of Arabic sweets and composite foods revealed that 23% and 51% are excellent sources of total dietary fiber whereas most of the remaining ones had only trace levels of it (Tables 1 and 2). Notably, nuts in Arabic sweets and stew recipes in traditional composite foods were identified as the primary sources of fiber. These results agree with studies on traditional South Asian cuisines in the UK and show higher fiber content than traditional dishes in Bahrain [36], Cameroon [37] and Italy [38, 39]. It has been noted that globally, people are commonly ingesting fewer than 20 g of DF daily (2,000 kcal/day for women and 2,600 kcal/day for men). Daily recommended dietary fiber intake is around 28 g/day for adult women and 36 g/day for adult men [40]. In Lebanon, the Dietary Fibers intake ranged between 14–19 g/day which fall below the daily recommendations (28g/2000 Kcal diet) [4].

**Table 1:** Total fibers, vitamin A, vitamin E, vitamin C content and the percentage of their daily contribution in 2000 Kcal-diet in 100 g and per serving size of traditional dishes.

Traditional dishes	Per 100 g of edible portions (unit /100g)				Percentage of daily contribution in 2000 Kcal			
	**Total Fibers** *	**Vit A** **	**Vit E** ***	**Vit C** ****	**Total Fibers**	**Vit A**	**Vit E**	**Vit C**
**Baba ghanouj** *(Aubergines)*	3.1	Tr	0.1	Tr	11	Tr	1	Tr
**Batata mahchi** *(Lamb ground with potatoes)*	1.8	Tr	Tr	Tr	6.4	Tr	Tr	Tr
**Borgul bi banadoura** (*Coarse bulgur wheat)*	4.2	Tr	1.5	Tr	15	Tr	10	Tr
**Chichbarak** *(Chichbarak Dough filled with ground beef and yogurt)*	Tr	Tr	Tr	Tr	Tr	Tr	Tr	Tr
**Falafel** (*Garbanzo beans)*	8.7	Tr	0.1	Tr	31	Tr	0.8	Tr
**Fatayer sabanikh** (*dough and Fresh spinach)*	2.1	Tr	0.1	Tr	7.5	Tr	0.7	Tr
**Fattat Hommos** (*Chickpeas)*	5.5	Tr	0.5	Tr	19.6	Tr	3.8	Tr
**Fattoush** (*Lettuces cherry tomatoes*, *cucumbers)*	6	Tr	0.1	7.2	21.4	Tr	0.8	8
**Foul moudamas** (*Broad beans)*	3.5	Tr	0.2	10	12.5	Tr	1.4	11.1
**Hindbe bil zet** (*Chicory greens*)	5.3	Tr	0.8	2.6	18.9	Tr	5.8	2.8
**Hommos bi tahini** (*Chickpeas)*	5.7	Tr	Tr	Tr	20.3	Tr	Tr	Tr
**Kafta wa batata** (*Minced lamb*, *potatoes)*	1.4	Tr	Tr	Tr	5	Tr	Tr	Tr
**Kebba bil sayniya** (*Finely ground beef)*	4.8	Tr	Tr	Tr	17.1	Tr	Tr	Tr
**Koussa mahchi** (*Minced lamb*, *small courgettes)*	1	Tr	Tr	Tr	3.5	Tr	Tr	Tr
**Lahm bil ajin** (*Plain white flour*, *minced lamb*)	1	Tr	Tr	Tr	3.5	Tr	Tr	Tr
**Loubia bil zet** (*green beans)*	1.9	Tr	0.5	2.6	6.7	Tr	3.9	2.8
**Malfouf mahchi** *(Cabbage leaves)*	1.3	Tr	Tr	Tr	4.6	Tr	Tr	Tr
**Moujadara** (*coral lentils)*	5.4	Tr	Tr	Tr	19.2	Tr	Tr	Tr
**Moghrabia** *(Dry dough)*	Tr	Tr	0.1	Tr	Tr	Tr	0.8	Tr
**Mousaka batinjan** (*Eggplant)*	3.5	Tr	0.2	5.6	12.5	Tr	1.9	6.2
**Riz a dajaj** *(Breast chicken*, *basmati rice)*	Tr	Tr	Tr	Tr	Tr	Tr	Tr	Tr
**Riz bi lahma** (*Medium fat meat*, *basmati rice)*	0.8	Tr	Tr	Tr	2.8	Tr	Tr	Tr
**Sayadia** (*Sea bass*, *basmati rice)*	Tr	Tr	Tr	Tr	Tr	Tr	Tr	Tr
**Shawarma dajaj** (*Chicken)*	Tr	Tr	0.9	Tr	Tr	Tr	6.4	Tr
**Shawarma lahma** (*Meat)*	Tr	Tr	Tr	3.8	Tr	Tr	Tr	4.2
**Tabboula** *(Tomatoes*, *parsley)*	3.2	Tr	0.1	21	11.4	Tr	0.8	23.3
**Warak enab** (*Vine leaves*, *rice*, *meat)*	6.7	Tr	Tr	Tr	23.9	Tr	Tr	Tr
**Yakhnat Bamia** *(Lamb cubed*, *okra)*	3	Tr	Tr	Tr	10.7	Tr	Tr	Tr
**Yakhnat Fassoulia** (*lamb*, *fresh white haricot beans*)	7.4	Tr	Tr	Tr	26.4	Tr	Tr	Tr
**Yakhnat Mouloukhia** (*Mouloukhiafree-range chicken*, *basmati rice*)	2	Tr	Tr	Tr	7.1	Tr	Tr	Tr

Tr: Trace-Low detection<0.01; Total Fibers—Total Fibers -Enzymatic-Gravimetric method; Vitamin A- High-Performance Liquid Chromatography; Vitamin E- High-Performance Liquid Chromatography; Vitamin C- Titrimetric, Dichloroindophenol method

*: Total fibers: g/100 g

**Vitamin A: mcg/100 g

***Vitamin E: mg/100 g

**** Vitamin C: mg/100 g

**Table 2:** Total fibers, vitamin A, vitamin E, vitamin C content and the percentage of their daily contribution in 2000 Kcal-diet in 100 g and per serving size of Arabic sweets.

Arabic sweet	In 100 g of edible portions (unit/100g)				Percentage of Daily Contributions in 2000 Kcal-diet			
	Total fibers*	Vit A**	Vit E***	Vit C****	Total fibers	Vit A	Vit E	Vit C
**Baklava mixed** *(Sheets of phyllo pastry*, *pistachios)*	11.8	8.1	1	2	42.1	0.9	6.6	2.2
**Baklava mixed light** *(Sheets of phyllo pastry pistachios)*	11.9	13	1.2	Tr	42.5	1.4	8	Tr
**Barazik** *(Sesame seeds*, *pistachios)*	4.1	Tr	Tr	8	14.6	Tr	Tr	8.8
**Boundoukia** *(Hazelnut)*	7.4	Tr	4.7	1	26.4	Tr	31.3	1.1
**Daoukia** (*Hulled unsalted pistachios*, *semolina)*	8.8	Tr	Tr	2	31.4	Tr	Tr	2.2
**Foustoukia** *(Egg white*, *almonds*, *pistachio)*	8.6	Tr	1.3	Tr	30.7	Tr	8.6	Tr
**Ghourayba** *(Organic sugar cane*, *egg yolks*, *butter at all-purpose flour*, *whole almonds)*	2.1	Tr	Tr	Tr	7.5	Tr	Tr	Tr
**Halawa** *(All-purpose flour*, *grounded aniseed*, *sesame seeds)*	3.9	Tr	Tr	Tr	13.9	Tr	Tr	Tr
**Halawa light** *(All-purpose flour*, *grounded aniseed*, *sesame seeds)*	3.4	Tr	Tr	Tr	12.1	Tr	Tr	Tr
**Halawat El jiben** (*Akkawi cheese*, *sugar*, *semolina)*	6.8	Tr	Tr	2	24.2	Tr	Tr	2.2
**Ish el bulbul** *(Kounafa dough*, *melted butter)*	10	8.4	0.1	3	35.7	0.9	0.7	3.3
**Kallaj kashta** (*Sugar*, *pistachios)*	4.6	Tr	Tr	Tr	16.4	Tr	Tr	Tr
**Karabij joz maa crema** (*Grounded finely walnuts*, *caster sugar*)	4	15.7	Tr	Tr	14.2	1.7	Tr	Tr
**Katayef Kashta** *(Flour*, *a pinch of salt*, *sugar)*	1.9	Tr	Tr	Tr	6.7	Tr	Tr	Tr
**Kounafa kashta bil kaak** *(Milk*, *cream fresh*, *hair pastry)*	3.1	Tr	Tr	Tr	11	Tr	Tr	Tr
**Kounafa bil jiben** *(Akkawi cheese or cow’s milk mozarella*, *"hair" pastry)*	3.1	Tr	Tr	Tr	11	Tr	Tr	Tr
**Maakaron** (*Blanched almonds plus whole almonds)*	NA	NA	NA	NA	NA	Tr	Tr	Tr
**Maakroun wa moushabak** *(Flour*, *corn flour*, *Sugar syrup)*	1.5	Tr	Tr	Tr	5.3	Tr	Tr	Tr
**Maamoul tamer** (*Semolina*, *grounded mahlab*, *clarified melted butter*,*)*	7	Tr	Tr	1.4	25	Tr	Tr	1.5
**Maamoul mad kashta** (*Semolina*, *ghee*, *sugar*, *fine semolina)*	8.8	Tr	Tr	Tr	31.4	Tr	Tr	Tr
**Maamoul mad joz** (*For filling*: *walnuts*, *pistachios*, *sugar Semolina*, *flour)*	3.9	13.4	2	2	13.9	1.4	13.3	2.2
**Maamoul fostok** *(Regular semolina*, *unbleached all-purpose flour*, *Hulled unsalted pistachio)*	6.2	12.7	Tr	Tr	22.1	1.4	Tr	Tr
**Maamoul joz** *(Semolina*, *grounded mahlab*, *walnuts)*	7.5	8.9	Tr	Tr	26.7	0.9	Tr	Tr
**Madlouka** (*Milk*, *starch*, *sugar*, *semolina*, *fried nuts almond and cashew)*	5.2	Tr	Tr	Tr	18.5	Tr	Tr	Tr
**Mafrouka kashta** *(Semolina*, *cream liquid milk*, *ground pistachio)*	2.8	Tr	Tr	Tr	10	Tr	Tr	Tr
**Mafrouka fostok** *(Sugar*, *butter*, *sweetened concentrated milk semolina*, *roasted pistachio mixture)*	NA	NA	NA	NA	NA	Tr	Tr	Tr
**Moufattaka** (*rice*, *sugar*, *tahini*, *turmeric)*	3	Tr	Tr	2	2	Tr	Tr	2.2
**Mouhallabiya** *(Whole milk*, *granulated sugar)*	1.2	Tr	Tr	Tr	Tr	Tr	Tr	Tr
**Moushabak** (*Flour*, *corn starch*, *sunflower oil*, *Sugar syrup)*	0.8	Tr	Tr	<0.1	Tr	Tr	Tr	Tr
**Nammoura** *(Regular semolina*, *fine semolina)*	8.4	Tr	Tr	<1	Tr	Tr	Tr	Tr
**Osmaliya** (*Kounafa packet*, *milk)*	2.5	Tr	Tr	<1	Tr	Tr	Tr	Tr
**Riz bil halib** (*Corn starch*, *full-fat milk*, *heavy cream)*	Tr	Tr	Tr	<1	Tr	Tr	Tr	Tr
**Saniora** *(Flour*, *sugar*, *ghee*, *butter*, *almonds)*	1.8	Tr	Tr	<1	Tr	Tr	Tr	Tr
**Sfouf** *(Semolina*, *flour)*	2.5	Tr	Tr	<1	Tr	Tr	Tr	Tr
**Shaaybiyat** (*Sheets of phyllo pastry*, *butter*, *fragrant sugar syrup*, *walnuts)*	1	Tr	Tr	<1	Tr	Tr	Tr	Tr
**Ward el sham** (*Phyllo pastry sheets*, *cream*, *butter*, *pistachios)*	2.3	Tr	Tr	<1	Tr	Tr	Tr	Tr
**Znoud El sitt** *(Spring roll dough*, *milk*, *liquid cream)*	2	Tr	Tr	<1	Tr	Tr	Tr	Tr

Tr: Trace- Low detection<0.01; NA: Not Available; Total Fibers -Enzymatic-Gravimetric method; Vitamin A- High-Performance Liquid Chromatography; Vitamin E- High-Performance Liquid Chromatography; Vitamin C- Titrimetric, Dichloroindophenol method.

*: Total fibers: g/100 g

**Vitamin A: mcg/100 g

***Vitamin E: mg/100 g

**** Vitamin C: mg/100 g

Dietary fibers are present in a variety of foods like cereals, fruits, nuts, seeds, legumes, and pulses.

Plant foods, especially whole grains and vegetables are highly rich in dietary fiber. Meat doesn’t provide dietary fiber whereas having a variety in dietary fiber-rich food items is very important. Incorporating DF from fruit and vegetable waste, (peels of grapes, cauliflower, pomegranates, lime and other citrus) improve the functional, nutritional and health attributes related to sustainable meat processing as they play the role of natural antioxidants. The process is explained by slowing down lipid oxidation and increasing shelf-life of meat products [40].

Plant foods have been linked to such health advantages as lower risk of obesity, cardiovascular disease (CVD) and some cancers. Fibers–types such as glucan and psyllium fibers are known to lower levels of cholesterol, increase insulin sensitivity, which may in turn prevent CVD diseases. They also have a laxative effect, which delays transit time, increases fecal bulk and fluid volume of stool, restricting the production of carcinogenic compounds in the colon and increasing fecal volume, which limits exposure time of intestinal mucosa to carcinogens. Moreover, Dietary fibers have a role in prevention of constipation and inflammatory bowel disease. Dietary fibers also stimulate the secretion of gut hormones, further contributing to regulation of satiety, gastrointestinal emptying and weight control. Research reveals an additional weight loss of 2.494 kg compared to other fiber types [40].

Furthermore, DF are crucial for the well-being of colon and cecum bacteria, dietary fibers are essential for the synthesis of short-chain fatty acids (SCFAs), which are critical for immune regulation and metabolism. Insufficient intake of fiber can result in decreased SCFA production, potentially affecting bodily functions. Furthermore, a high-protein diet can trigger the formation of harmful amino acid metabolites, potentially worsening chronic diseases [41]. Our investigation revealed that numerous traditional meals had low total fiber content, likely due to cooking methods like vegetable peeling that reduce soluble fiber levels. Cooking procedures can also reduce the amount of dietary fiber present, affecting both soluble and insoluble fiber. However, Cooking techniques like boiling and roasting have been proven to increase overall dietary fiber content through the creation of fiber-protein complexes [41].

#### Vitamin A, D, E and C

The analysis of various traditional composite dishes and Arabic sweets revealed in this study, the presence of trace amounts of vitamins A, D, E, and C (Table 1). The concentration of vitamin A differed among specific sweets, with levels ranging from 8ug to 15ug of 100 g of edible foods. However, it is noteworthy that all these sweets contained less than 5% of the daily recommended intake of vitamin A. While most traditional foods contained traces of vitamin C, tabbouleh stood out as the only dish that offered more than 20% of the daily recommended intake of vitamin C.

The micronutrient composition of Arabic treats has not been widely explored beyond Bahrain. Our study aligns with the findings of Musaiger et al., highlighting the inadequate levels of vitamins A, D, E, and C in Arabic sweets from Lebanon and Bahrain [36].

Our research on mixed dishes showed decreased levels of vitamin A in comparison to the amounts found in four traditional dishes from Cameroon [37]. These findings are consistent with studies conducted in Italy [38] and Bahrain [36] which also indicated that traditional dishes generally lack vitamin A. The topic of vitamin A consumption has been extensively debated in various Arab nations. For instance, inadequate intake of vitamin A has been observed among children in Egypt and Sudan [42, 43]. Moreover, in Jordan, half of Bedouin children aged 6–66 months are vulnerable to vitamin A deficiency [44]. Similarly, around 70% of children between the ages of 4 and 13 in Lebanon suffer from vitamin A deficiencies due to their diet [22]. This issue is also common among adolescents aged 17–19 [45], as well as adults aged 20 and older [4]. Improving the availability of foods abundant in vitamin A is imperative to enhance the nutritional status and growth of malnourished communities where vitamin A deficiency poses a concern. Primary sources of vitamin A encompass dairy products, eggs, fish, and liver. Considerable amounts can also be found in fruits, leafy green vegetables, orange and yellow vegetables, tomato products, and specific vegetable oils. Moreover, certain foods like milk and margarine are often fortified with extra vitamin A. To enhance their nutritional content, some ready-to-eat cereals are fortified with vitamin A. In low-income countries, plant-based foods play a crucial role in providing this essential nutrient. The process of cooking or heating specific foods can boost the levels of beta-carotene, which serves as a precursor to vitamin A [46–48]. In relation to vitamin D, minimal quantities were detected in all composite dish samples except for "Sayadia" (rice and fish), which contained 3.2 g of vitamin D per 100 g. This amount accounted for 21% of an adult’s daily vitamin D requirement (data not reported in Table 1). The findings are consistent with the study carried out by Spearing et al., indicating that the traditional foods eaten in Empangeni, South Africa, generally do not contain enough vitamin D, except for the well-known "fish stew" [49]. Likewise, an examination of the cultural food preferences of South Asian dishes in the UK revealed that fish curries were distinguished by their particularly rich vitamin D levels [39]. Nutritional evaluations of traditional foods in specific Arab Gulf nations have been investigated [42]. Several mixed dishes commonly eaten in Saudi Arabia have been identified as lacking in vitamin D [49]. Lebanese and Saudi Arabian traditional foods display deficiencies in vitamin D, particularly in dishes like Hummus, Foul Mdames, Warak Inab, and Moussakaet Batenjen. Nevertheless, Fatayer Sabanekh is notable for its high vitamin D content (75.88 IU/100 g), followed by Batata Mihchi (18.99 IU/100 g), both of which originate from Saudi Arabia, when compared to their Lebanese equivalents. This could be attributed to the use of vitamin D fortified margarines in cooking. Our study on falafel (1.77 IU/100 g) from Saudi Arabia supports the findings of Al Faris et al., who found that falafel has limited amounts of Vitamin D [50]. Similarly, Lebanon, like other Arab countries, faces a widespread deficiency of vitamin D among individuals of all ages due to insufficient dietary sources [51]. The literature extensively discusses the fluctuations in vitamin D levels that are related to seasonal changes, primarily influenced by exposure to sunlight. Additionally, an association exists between Percent Body Fat (PBF), waist circumference and inadequate levels of vitamin D. People with lower vitamin D levels tend to show significantly increased percentages of body fat and higher waist circumference [52].

Due to conservative attire and limited availability of vitamin D-rich foods, the MENA region faces challenges with low blood 25-hydroxyvitamin D (25(OH) D) levels [26, 53]. This deficiency affects 46–83% of adolescents and adults in the MENA region [49]. Research has shown a correlation between low vitamin D levels and various health conditions, including cardiometabolic disorders, osteoporosis, rickets, and osteomalacia in infants and toddlers, as well as osteomalacia in adults [54]. Moreover, the concurrence of hypovitaminosis D and metabolic syndrome is common, since deficient vitamin D levels can exacerbate insulin resistance, thereby serving as the connecting factor among different components of metabolic syndrome [55]. Besides, recent studies showed that vitamin D and its derivatives, along with other minerals, act as immunomodulators as they have a role in respiratory illnesses, while specifically delineating the plausibility of employing them as a preventive and therapeutic agent against pandemics like COVID-19 from an immunological perspective. A lack of vitamin D not only can raise the chances of getting severely ill with Covid-19, but also cause inflammation associated with existing pandemic viral infections. Studies showed that insufficiency of vitamin D can increase the severity of it. Whereas the treatment with vitamin D attenuates the inflammation caused by the viral infection.

Thus, the high-risk profile of COVID-19 is associated with vitamin D deficiency, and this in turn is influenced by ethnicity, geography, and seasonal changes as well as measure frequency. A study conducted in India also showed that hypo-vitaminosis was linked to COVID-19 and not the normal state in 156 patients of COVID-19 positive and 204 controls as well. Compared to the controls, the COVID-19 patients had a serum vitamin D level less than 10 ng/mL in the study [56].

The analysis revealed that most of the foods lacked sufficient vitamin E content, apart from Borgul bi banadoura, which contained a notable amount of the vitamin, providing 10% of the daily requirement (see Table 1). However, the absence of information on vitamin E composition and intake limits our ability to compare these findings with national or regional data. This lack of data could be attributed to the infrequency of vitamin E deficiency, particularly in developed countries. Conversely, in developing countries, vitamin E deficiency is common among children58].

Most traditional dishes typically lack significant amounts of vitamin C. Tabboula, on the other hand, is the exception as it provides more than 40% of the daily recommended intake for this essential nutrient (refer to Table 1). Similar findings have been reported in the United Kingdom [39], South Africa [49], and Bahrain [36]. The inadequate consumption of vitamin C from food was observed nationwide across all age groups [46].

The limited availability of foods rich in vitamin C and the resulting deficiency among populations can be attributed to several factors. Firstly, staple foods such as grains generally have low levels of vitamin C. Secondly, the vitamin content in raw vegetables can vary depending on whether they are fresh or frozen, as well as other factors like cultivar conditions. Cooking processes, especially prolonged boiling and blanching, can cause significant degradation of vitamin C. However, methods like microwaving and steaming, which involve shorter cooking times and less water, are more effective in retaining the nutrient [57]. The cooking techniques and processing steps used in traditional Lebanese dishes like Meloukhieh, Cauliflower Stew, and Aadas Bhamoud significantly impact vitamin C content. Research findings reveal that there is a noticeable decrease in vitamin C levels during processing. In fact, reductions of 37.64%, 65.43%, and 79.00% were observed in Meloukhieh, Cauliflower Stew, and Aadas Bhamoud, respectively. Furthermore, the choice of cookware also plays a role, as Double Based Stainless Steel (DBSS) shows less depletion compared to Pressure Cookers (PCs). Additionally, refrigeration affects the vitamin C levels in Aadas Bhamoud and Meloukhieh, while reheating leads to significant vitamin C losses across all dishes and reheating methods [58].

It is crucial to investigate the vitamin C levels in traditional foods because of its scarcity, considering the potential negative health impacts of vitamin C deficiency like anemia and bleeding sores. The combination of these findings, which indicate poor consumption and limited availability of micronutrients, is concerning since it raises the possibility of malnutrition and should be a top priority for policymakers. Moreover, cooking causes meals to lose some of their vitamins and minerals. During processing and cooking, vitamins and minerals may be lost by oxidation or dissolution in water. Foods that are processed may also lose nutrients due to their sensitivity to heat, light, oxygen, solvent pH, or a combination of these factors [59].

Nutrient loss can also happen during storage, handling in the home and business, catering, and the time between harvest and delivery. In many countries, epidemiological and dietary studies that potentially result in micronutrient deficiencies consider the impact of cooking losses on vitamin and mineral consumption. However, the nutritional content and composition of nutrients have been seldom examined in relation to food preparation and cooking methods [60].

### Limitations

The primary constraint of this study pertains to the analysis of nutrients and minerals within a limited selection of Lebanese traditional dishes. Furthermore, as the food samples were acquired from central kitchens, the exact proportions of ingredients used were not specified. The ingredients themselves were sourced from literature references. Additionally, variations in ingredients and preparation methods among traditional dishes from different governorates in Lebanon were inevitable.

## Conclusion

This study sheds light on the nutritional composition of traditional Lebanese dishes and Arabic sweets, in terms of total dietary fibers and vitamins, underscoring their role in maintaining a well-balanced diet.

Although a significant part of these foods enclose specific amount of fiber (especially in traditional meals), however the overall presence of essential vitamins such as vitamins A, D, E and C is quite poor. Most Arabic sweets are particularly low in these vitamins, with variable levels only seen in a few foods for vitamin A. As for traditional meals, they provide marginal micronutritional values, except for Tabboula as a source of vitamin C. This study showed that both Lebanese foods and Arabic sweets lack key micronutrients. This holds the importance of adding more rich nutrients in Lebanese diet to meet all essential vitamins and fibers. By examining key micronutrients and total dietary fibers, valuable insights are gained to address health issues in Lebanon. Therefore, knowing the nutrient composition of traditional meals may help in translating ways to introduce healthier cooking techniques, or ingredient changes which respect cultural identity while providing positive health outcomes. Nutritional deficiencies are a major health concern in a population and have growth and development consequences especially when paired with overnutrition. Promoting a healthier diet, can help prevent nutrition-related non-communicable diseases such as diabetes and cardiovascular diseases. Moreover, the study emphasizes the significance of preserving culinary heritage while promoting nutritional diversity. This is important as traditional diets form the cornerstone of cultural identity and knowing what they mean nutritionally would be critical for programming balanced eating in communities that have deep connections to these dishes. In alignment with Sustainable Development Goal number two, it proposes interventions such as biofortification, reducing food waste, enhancing food processing education, and adopting a holistic approach from farm to plate. Establishing strong collaborations between agriculture, nutrition, health sectors, and policymakers is essential for sustainable solutions to malnutrition in the long run.

## Supporting information

S1 Table Specifications related to food handling. (DOCX)

S2 Table Ingredients related to composite dishes. (DOCX)

S3 Table Ingredients related to Arabic sweets. (DOCX)

S4 Table Chemical analysis of Vitamin A, E, C, D and total fibers. (DOCX)
