# Supplementary material for: The dietary fiber and micronutrient composition of traditional foods from Lebanon and their contribution to dietary adequacy: A call for action
Source: PLoS One. 2024 Oct 29;19(10):e0312429. doi: 10.1371/journal.pone.0312429 (PMC11521292; doi:10.1371/journal.pone.0312429)
Supplement: S1 Table — (DOCX) [file pone.0312429.s001.docx]

| **S1 Table. Specifications related to Food handling** | | | | | |
| --- | --- | --- | --- | --- | --- |
|  | **Temperature** | **Time of Handling** | **Storage** | **Transportation** | **Containers** |
|  |  |  |  |  |  |
| **Total Fibers** *(Enzymatic-Gravimetric method)* | **Incubation Temperature**  **Alpha-amylase:** Sample incubation (95-100°C for 30 minutes)  **Protease:** Sample incubation (60°C for 30 minutes)  **Amyloglucosidase:** Incubate at Sample incubation (60°C for 30 minutes)   **Room Temperature** Filtration and weighing, at standard room temperature (20-25°C). | **Sample Preparation** Minimize the time between sample collection and analysis  **Enzymatic Reactions:** Adhere strictly to the specified incubation times for each enzymatic step   **Filtration and Drying** Directly filter and dry the residue after enzymatic digestion | **Sample Storage** Store samples in a cool, dry place, away from direct sunlight and moisture, in a desiccator **Reagents and Enzymes:** Store enzymes and reagents in a refrigerator or freezer  **Post-Analysis Residues** Store dried residues in a desiccator until weighing. | **Sample transportation** Transport samples in airtight, moisture-proof containers. Use insulated containers. **Reagents and Enzymes** Transport enzymes and reagents in cold storage (insulated cooler with ice packs) | **Sample Containers** Use airtight, moisture-proof containers (glass or plastic)   **Filtration Equipment:** Use fritted glass filters suitable for the sample matrix and resistant to the reagents used.  **Desiccators:** Use desiccators with appropriate desiccant material (silica gel). |
|  |  |  |  |  |  |
|  |  |  |  |  |  |
|  |  |  |  |  |  |
|  |  |  |  |  |  |
|  |  |  |  |  |  |
|  | | | | | |
| **Vitamin A** *(High-Performance  Liquid Chromatography***)** | **Analysis Temperature  Column Temperature**: 25-30°C.   **Storage Temperature Vitamin A Standard Solutions:** Should be stored in amber glass containers at -20°C.  **Sample Storage:** Samples should be kept at <-20°C. | **Sample Preparation and analysis**  Minimize the time samples are at room temperature  Prepare standards and samples directly before analysis  **Stability** Perform all handling in subdued light and minimize exposure to air. Once prepared, samples are directly injected into the HPLC system or are stored at low temperatures (freezer) | **Vitamin A Solutions** Store in tightly sealed amber glass containers at -20°C and avoid exposure to light by wrapping containers in aluminum foil or using opaque containers.  **Solid Samples** Store in a dry, cool, and dark place, under inert gas (like nitrogen) If samples are in solution form, freeze them to avoid degradation | **Temperature-Controlled transportation** Use insulated containers with ice packs to maintain low temperatures during transportation. **Light Protection** Use opaque containers or wrap transparent containers in aluminum foil | **Material** Use amber glass containers for storage and handling.  For sample collection, use glass or amber polypropylene or high-density polyethylene (HDPE) containers**.** |
|  |  |  |  |  |  |
|  |  |  |  |  |  |
|  |  |  |  |  |  |
|  |  |  |  |  |  |
|  |  |  |  |  |  |
|  | | | | | |
| **Vitamin D** *(High-Performance  Liquid Chromatography)* | Samples are prepared at room temperature (around 20-25°C) or under refrigeration (4°C) . HPLC analysis is performed at slightly elevated temperatures (25-40°C) | Minimize the time between sample preparation and injection into the HPLC system | Samples are stored in amber-colored vials and at low temperatures | Samples are well packed and shipped | Amber-Colored Vials: Samples and standard solutions stored in amber-colored vials |
|  |  |  |  |  |  |
|  |  |  |  |  |  |
|  | | | | | |
| **Vitamin E** *(High-Performance  Liquid Chromatography)* | Samples are kept on ice, and temperature is optimized to detect and separate Vitamin E. | Minimize exposure of the samples to light, air, and heat during handling. | Samples are stored in opaque and airtight containers. | Samples are transported in insulated containers or coolers with ice packs, especially for samples that require refrigeration or freezing. | Glass or inert plastic containers are used for HPLC analysis. |
|  |  |  |  |  |  |
|  |  |  |  |  |  |
|  | | | | | |
| **Vitamin C** *(Titrimetric, Dichloroindophenol method)* | Samples are titrated around 20-25°C. | Minimize exposure to light and air during handling. | Samples should be stored in airtight containers. | Samples are transported in insulated containers | Glass or inert plastic containers are used. |
|  |  |  |  |  |  |
|  |  |  |  |  |  |
|  |  |  |  |  |  |
|  | | | | | |
