# Supplementary material for: The dietary fiber and micronutrient composition of traditional foods from Lebanon and their contribution to dietary adequacy: A call for action
Source: PLoS One. 2024 Oct 29;19(10):e0312429. doi: 10.1371/journal.pone.0312429 (PMC11521292; doi:10.1371/journal.pone.0312429)
Supplement: S2 Table — (DOCX) [file pone.0312429.s002.docx]

S2 Table. Ingredients related to composite dishes.

| **Dish** | **Ingredients** |
| --- | --- |
| Baba ghanouj | Aubergines, garlic cloves, juice of lemon, tahini, pomegranate seeds, salt |
| Batata mahchi | Lamb ground onions, butter, salt, pepper, pine nuts, potato, tomato juice |
| Borgul bi banadoura | Coarse bulgur wheat, small pearl onions, chickpeas, cinnamon stick, caraway seed, vegetable oil, mild white pepper, salt |
| Chichbarak | Chichbarak Dough: multi-purpose flour, salt, water warm to form a paste, yeast, sugar Meat Stuffing: ground beef, salt to taste, black pepper to taste, cinnamon powder to taste, onion finely chopped, pine nuts, olive oil, bushel of parsley chopped Chich Barak Stew: yogurt, water, starch, garlic cloves crushed (optional), rice, dried mint, salt to taste |
| Falafel | Dry peeled fava beans dried chickpeas (Garbanzo beans), Italian parsley (chop away the stems), green cilantro (chop away the stems), freshly peeled crushed garlic cloves, red or yellow onion, green onions, salt, black pepper, flour, baking soda, red chili pepper (optional, if spicy falafel is desired), cumin Coriander. Falafel Tahini Sauce Ingredients: Tahini Paste, freshly squeezed lemon juice, garlic cloves, crushed, salt |
| Fatayer sabanikh | Fresh spinach, onions, pine nuts, lemon juice, olive oil, sumac, salt, plain white flour, caster sugar, baker yeast, olive oil, salt |
| Fattat Hommos | Chickpeas, tomatoes, onion, basil leaves, garlic cloves, pitta bread, pine nuts, yogurt, tahini and vinegar, vegetable oil, salt and pepper |
| Fattoush | Lettuces or romaine lettuce, cherry tomatoes, cucumbers, radishes, spring onions, flat-leaf parsley mint, pitta bread, olive oil, vinegar, sumac, salt |
| Foul moudamas | Broad beans, baking soda, water, water, salt, garlic cloves minced, lemon juice, olive oil |
| Hindbe bil zet | Chicory greens, water, olive oil, onions, salt, lemon juice |
| Hommos bi tahini | Chickpeas, garlic cloves, lemon juice, tahini, olive oil, salt |
| Kafta wa batata | Minced lamb, flat-leaf parsley, onions, salt and pepper, onions, red pepper, tomato juice, debs roman, olive oil, salt and pepper, potatoes, ripe tomatoes, vegetable oil. |
| Kebba bil sayniya | FInely ground beef (or lamb, lean, divided), bulgur cracked wheat, salt, all spice, cumin, onions (finely chopped) |
| Koussa mahchi | Minced lamb, small courgettes, short grain rice, olive oil, salt and black pepper |
| Lahm bil ajin | Plain white flour, caster sugar, baker’s yeast, salt, olive oil, minced lamb, tomatoes, few drops of pomegranate molasses, salt and pepper |
| Loubia bil zet | Vegetable oil, white onions, sliced, Frozen bag green beans, garlic cloves, peeled, Cans of chopped tomatoes, Salt and sweet pepper, to taste, 7 spices, Extra-virgin olive oil |
| Malfouf mahchi | Cabbage leaves, basic vegetables stuffing, tomato, lemon juice, water, cinnamon, garlic cloves, dry mint |
| Moujadara | Green or coral lentils, short-grain rice, onions, olive oil, salt |
| Moghrabia | Dry dough, Chick Peas, Pearl Onions, Vegetable Oil , Butter Caraway, Ground Cinnamon Ground Cumin, salt, black pepper |
| Mousaka batinjan | Eggplant, sliced into (you can leave skin on or peel), yellow or white onion, diced, garlic cloves, minced, low-salt chickpeas, extra virgin olive oil, low-salt diced tomatoes, tomato paste, Piquant Post Spicy Mint blend, Pita chips or crusty bread for dipping, salt and pepper to taste |
| Riz a dajaj | Breast chicken, basmati rice, carrot, onions, tomato juice, whole black peppercorns, whole green cardamoms, cinnamon, cloves, cumin, vegetable oils, salt |
| Riz bi lahma | Medium fat meat, basmati rice, carrot, onions, tomato juice, whole black peppercorns, whole green cardamoms, cinnamon, cloves, cumin, vegetable oils, salt |
| Sayadia | Sea bass, scaled and gutted or in fillets, basmati rice, onions, caraway seeds, ground cumin, pines nuts, olive oil, fish stock, vegetable oil, salt, flour, butter, lemon juice |
| Shawarma dajaj | Chicken, olive oil, onions, red vinegar, lemon juice, pepper, cinnamon, nutmeg, salt, 6 cloves of garlic |
| Shawarma lahma | Meat, olive oil, onions, red vinegar, lemon juice, pepper, cinnamon, nutmeg, salt, 6 cloves of garlic |
| Tabboula | Tomatoes, spring onions, flat leaf parsley, mint, bulgur, wheat, lemon juice, olive oil, salt |
| Warak enab | Vine leaves, tomatoes, onion, flat-leaf parsley, mint, lemon juice, short-grain rice, meat, olive oil, salt |
| Yakhnat Bamia | Lamb cubed, onions, garlic cloves minced, green coriander, okra, lemon juice, salt, pepper, water and tomatoes |
| Yakhnat Fassoulia | Shoulder of lamb, fresh white haricot beans, coriander, onions, garlic cloves, tomato juice, olive oils, 500 ml water or chicken stock, salt and black pepper |
| Yakhnat Mouloukhia | Mouloukhia, free-range chicken, basmati rice, coriander, garlic cloves, onion, shallots, pitta bread, vinegar, juice of lemons, vegetable oil and salt |
