# Supplementary material for: The dietary fiber and micronutrient composition of traditional foods from Lebanon and their contribution to dietary adequacy: A call for action
Source: PLoS One. 2024 Oct 29;19(10):e0312429. doi: 10.1371/journal.pone.0312429 (PMC11521292; doi:10.1371/journal.pone.0312429)
Supplement: S3 Table — (DOCX) [file pone.0312429.s003.docx]

S3 Table. Ingredients related to Arabic Sweets.

| **Arabic sweet** | **Ingredients** |
| --- | --- |
| Baklava Mixed | Sheets of phyllo pastry, unsalted melted butter, fragrant sugar syrup. Filling Ingredients: hulled unsalted pistachios, superfine sugar, orange blossom water, rose water. |
| Baklava Mixed Light | Sheets of phyllo pastry, unsalted melted butter, Sugar Alcohol. Filling Ingredients: hulled unsalted pistachios, Sugar Alcohol, orange blossom water, rose water. |
| Barazik | Sesame seeds (lightly toasted), clarified chilled butter, icing sugar, egg, vanilla, vinegar, flour, baking powder, a pinch of salt, thinly sliced pistachios, milk. |
| Boundoukia | Hazelnut, sugar, water, corn flour, salt, orange blossom water, rose water, butter |
| Daoukia | Hulled unsalted pistachios, semolina, sugar, milk, orange blossom water, rose water, butter, green colorant. |
| Foustoukia | Egg white, fine sugar, fine powdered sugar, blossom water, almonds, pistachio, dried flowers for decoration. |
| Ghourayba | Organic sugar cane, small grains mastic to yield powdered mastic, egg yolks, unsalted butter at room temperature, unbleached all-purpose flour, blanched whole almonds. |
| Halawa | Unbleached all-purpose flour, grounded aniseed, grounded cinnamon, small grains mastic finely grounded to yield powdered mastic, sesame seeds, blanched toasted almonds, confectioners' sugar, honey, unsalted butter, sunflower oil. |
| Halawa light | Unbleached all-purpose flour, grounded aniseed, grounded cinnamon, small grains mastic finely grounded to yield powdered mastic, sesame seeds, blanched toasted almonds, Sugar alcohol, unsalted butter, sunflower oil. |
| Halawat El Jiben | Akkawi cheese, sugar, semolina, water, arabia kashta, orange blossom water, rose water, sugar syrup, lemon blossom and grated pistachio for decoration. |
| Ish el bulbul | Kounafa dough, melted butter, honey, kashta, sweetened lemon blossom, pistachios. |
| Kallaj kashta | Sugar, pistachios, lemon blossom, milk, kallaj sheets. Kashta ingredients: milk, cream fresh, rose water, blossom water, corn flour, sugar. Sugar syrup ingredients: sugar, water, lemon juice |
| Karabij joz maa crema | Grounded finely walnuts, caster sugar, cold water, rose water, extra- fine semolina, unsalted softened butter, granulated sugar, grounded  mahlab, whole milk, active dry yeast dissolved with sugar in water. Cream Ingredients: egg whites, sugar, rose water, blossom water |
| Katayef kashta | Flour, a pinch of salt, sugar, instant dry yeast, lukewarm water, baking soda, rose syrup. Akkawi cheese or ricotta mixed with mozarella, fresh grated mozarella cheese, sugar, rose water. |
| Kounafa kashta maa kaak | Milk, cream fresh, rose water, blossom water, corn flour, sugar, "hair" pastry, unsalted diced butter, fragrant sugar syrup. |
| Kounafa bil jiben | Akkawi cheese or cow's milk mozarella, "hair" pastry, unsalted diced butter, fragrant sugar syrup. |
| Maakaron | Blanched almonds plus whole almonds, superfine sugar, almond extract, freshly squeezed lemon juice, egg whites. |
| Maakroun wa moushabak | Flour, corn flour, yeast, water. Sugar syrup: sugar, water, lemon juice |
