# Supplementary material for: The dietary fiber and micronutrient composition of traditional foods from Lebanon and their contribution to dietary adequacy: A call for action
Source: PLoS One. 2024 Oct 29;19(10):e0312429. doi: 10.1371/journal.pone.0312429 (PMC11521292; doi:10.1371/journal.pone.0312429)
Supplement: S4 Table — (DOCX) [file pone.0312429.s004.docx]

S4 Table. Chemical Analysis of Vitamin A, C, D, E and Total Fibers.

|  | **Reagent preparation** | **Assay Procedure** | **Sample Analysis and units** |
| --- | --- | --- | --- |
|  |  |  |  |
| **VitaminC** *(Titrimetric, Dichloroindophenol method)* | **Reagents:**  - Phosphorous acid,  -HPO₃ glacial sublime: 15 g  -Glacial acetic acid (CH₃COOH): 40mL  -Distilled H₂O: 150 mL  Procedure:  1- Weigh by means of an analytical balance 15 g of glacial HPO₃.  2- In a glass screw-capped vail, add 40 mL glacial CH3COOH.  3- Dissolve the 15 g of HPO₃ in the acetic acid and stir until good dissolving.  4-Mix and add 150 ml H₂O.  5- In 250mL volumetric flask add distilled H₂O to the mark.  6- Filter the solution into a beaker (500 mL) to remove any undissolved particles.  **Preparation of EDTA Solution:**  **Reagents**:  -0.9 g of ethylenediaminetetraacetic acid (EDTA)  -Distilled H₂O: 200 mL  **Procedure:**  1-Weigh 0.9 g of EDTA.  2-Put 200 mL distilled H2O in a container and dissolve the EDTA in it.  3-Transfer into a 250 mL volumetric flask after that fill to the mark with distilled H₂O.  **Preparation of Ascorbic acid standard solution**  **Reagents:**  Ascorbic acid standard solution preparation:  Reagents:  -Ascorbic acid: 50 mg  -Same as that for sample preparation: up to 50 ml of Precipitant solution.  **Procedure:**  1- Accurately weigh, 50 mg ascorbic acid  2- Put the needed agent in a 50 mL volumetric flask and fill to volume (1 mg/mL with of 50 mg of precipitant in 50 mL).  **Preparation of Indophenol Standard Solution:**  **Reagents:**  -2,6-Dichloroindophenol Na salt: 0.0625 g  -Sodium bicarbonate (NaHCO₃): 0.0525 g  -Distilled H₂O: 50 mL, then diluted to 250 mL  **Procedure:**  1-Add 0.0625 g 2,6-dichloroindophenol Na salt and 0.0525 g NaHCO₃  2- Dissolve both the compounds in 50ml distilled water.  3- Quantitatively transfer this into a 250 mL volumetric flask and make up to the mark with distilled water.  4-Drain the solution and store it in the fridge. | **Titration Setup:**  -Use three 50 mL Erlenmeyer flasks  -With increasing the amount of the precipitant solution, 5 mL for each flask is added.  **Ascorbic Acid Titration**:  **Procedure**:  -Pipet 2.0 mL of the ascorbic acid standard solution to each flask.  - Titrate each flask with the indophenol standard solution.  -A faint rose-pink color that lasts for 5 seconds, is the end point of titration.  **Preparation of Blank Samples:**  **Procedure:**  -Add the stunt solution in 3 Erlenmeyer, by putting 7 mL of the solution, and then add 15 mL distilled H₂O.  -Titrate these blank solutions with indophenol standard solution. | **Sample Preparation:**  **Reagents:**  Sample: 5 g (solid sample)  Precipitant solution: 50 mL  Procedure:  -Accurately weigh 5 g of sample.  -Place the sample in 50 mL of precipitant solution in a 125 mL beaker  -Add the well mixed solution and stir it for 10 mins perfectly dissolved.  -Filter the solution to extract any undissolved components.  **Aliquot Division:**  Distribute filtered solution into three 50 mL Erlenmeyer flasks.  Volume of each aliquot (5–20 mL; volume depends on approximate concentration of vitamin C).  **Titration of Samples:**  **Procedure:**  Follow the same procedures as described above for an ascorbic acid standard to titrate each aliquot with the indophenol reference solution.  Also prepare two blank samples as before for positive control.  **Units:**  Mass is measured in grams (g).  Volume is measured in milliliters (mL).  Concentration may be reported in grams per liter (g/L) or milligrams per milliliter (mg/mL). |
|  |  |  |  |
|  |  |  |  |
|  |  |  |  |
|  |  |  |  |
|  |  |  |  |
| **VitaminA** *(High-Performance  Liquid Chromatography***)** | **Sample Preparation:**  For each replicate, homogenized samples of plant material (2 g to 10 g) were weighed.  Each sample was paired with the following:  -1 g of ascorbic acid (C₆H₈O₆)  -150 mL of ethanol (C₂H₅OH)  -A 60% (w/v) KOH solution was diluted in enough water to volume of 100 mL with final concentration of 50 mL. | **Saponification:**  -The extract obtained was transferred to an Erlenmeyer flask capacity of 250 mL and protected from the light.  -The flask was stirred continuously overnight (~16–24 hours) at room temperature (~20–25 °C), until complete saponification.  **Extraction:**  -The reaction mixture obtained after saponification was extracted in a separatory funnel.  -Add 150 mL of H2O with an accuracy of ±0.1 mL to the amount of water suitable for this solution concentration.  -The mixture was mixed well and let to seclude. After three extractions with 100-mL portions of hexane (C₆H₁₄), the organic phase was collected and dried over anhydrous Na₂SO₄.  **Washing and Drying:**  -The hexane extracts from the samples were each collected and washed with distilled water (50 mL) to remove any residual polar compounds.  -The organic layer was dried by adding 10 g anhydrous sodium sulfate (Na₂SO₄) and allowed to stand for 30 min for hydration.  **Evaporation:**  After that, the hexane was evaporated by rotary evaporator (rotation at 40°C and reduced pressure) to leave target compounds in form of residue.  **Sample Dissolution:**  The dried residue was then dissolved in 10 mL of a 2% (v/v) isopropanol (C₃H₈O) in hexane solution. | **Chromatographic Analysis:**  -Appropriate HPLC was utilized to analyze the prepared sample.  -A Lichosorb Si 60 column was used (250 mm ×4.6 mm, 5 µm particle), with a mobile phase of hexane and isopropanol at the flow rate = 1 mL/min.  -The injection volume was 20 µL.  **Calibration Curve:**  -Standards were used to establish a calibration curve at different known concentrations (0. 1, 0.5, 1.0, 5.0 and 10 mg/mL etc).  -A given AUC for each standard peak was recorded, and linear regression analysis was performed to correlate peak area of a compound to concentration.  -The concentration of samples was calculated by the peak area at the same retention time of standard.  Units:  Mass is measured in grams (g).  Volume is measured in milliliters (mL).  Concentration may be reported in grams per liter (g/L) or milligrams per milliliter (mg/mL). |
|  |  |  |  |
|  |  |  |  |
|  |  |  |  |
|  |  |  |  |
|  |  |  |  |
| **VitaminD** *(High-Performance  Liquid Chromatography)* | **Samples:** Homogenized plant or biological material (10–30 g)  **Reagents:**  -Pyrogallol (1 g)  -Ethanol (100 mL, absolute or 95% ethanol)  -Potassium hydroxide (KOH) solution (50 mL of 50% KOH, w/v)  -Hexane (300 mL total for extractions)  -Water (H2O, 300 mL total for washing and extractions)  -Anhydrous sodium sulfate (Na2SO4) for drying the organic layer  -Butylated hydroxytoluene (BHT) for antioxidant protection  -Methanol for dissolving the residue  **Equipment**:  -Homogenizer  -Separatory funnel  -Rotary evaporator or similar for solvent evaporation  -C18 chromatography column  -UV spectrophotometer (for detection at 265 nm) | **Procedure**  **Sample Preparation**:  -Weigh the homogenized sample (10 g — 30 g) with an analytical balance and record that exact weight, to perform calculations.  **Saponification:**  Combine the following in an appropriate reaction vessel:  -Homogenized sample (10–30 g)  -Pyrogallol (1 g)  -Ethanol (100 mL)  -50% KOH solution (50 mL)  Nitrogen Environment Method:  1-Purge the vessel to obtain an inert atmosphere.  2-Heat to 70–100 ° C for 20–45 min.  3-*Room Temperature Method*:  Cover the vessel in aluminum foil to block out light.  -Keep the mixture stirred at room temperature for 12 hours  **Saponification processing steps:**  - Pour the reaction mixture into a separatory funnel.  -Transfer funnel to a new container and add 150 mL of H2O, mixing gently for complete phase separation.  Extraction:  -Conduct three hexane extractions: Extract with 100 mL of hexane.  -Next, shake the separatory funnel very well and then separate the phases.  -Each organic phase was collected separately into a different container.  -At the last extraction, wash the organic phase with 3 x 100mL H2O to remove remaining water-soluble components. | **Drying and Evaporation**:  -Add the organic phase to a drying flask, add anhydrous sodium sulfate (Na2SO4) if trace water is still present.  -Sodium sulfate is filtered off from solution.  -Use a rotary evaporator to evaporate the filtered hexane under reduced pressure until just a solid residue remains.  **Antioxidant Addition:**  -To the dried residue, add BHT at final concentration 0.1% (w/v) to prevent oxidation during storage.  Dissolution for Analysis:  Add 10 ml to dissolve the residue for chromatographic analysis.  **Chromatographic Analysis:**  -Chromatographic separation was carried out using a C18 column.  -The mobile phase was methanol with UV detection at 265 nm.  -Calibration solutions with known concentrations are then injected so a calibration curve can be constructed.  **Calibration Curve and Sample Analysis**  **-**Prepare standard solutions with various concentrations known (0, 10, 20 to 30 and 50 ug/ml)  -Each standard solution was injected, and the peak areas recorded.  -Use the calibration curve built from peak area versus concentration.  -Inject sample solutions and calculate the concentrations in them by linear interpolation of their peak areas with a calibration curve.  **Units:**  Mass is measured in grams (g).  Volume is measured in milliliters (mL).  Concentration may be reported in grams per liter (g/L) or milligrams per milliliter (mg/mL). |
|  |  |  |  |
|  |  |  |  |
| VitaminE (High-Performance  Liquid Chromatography) | **Materials**  -Sample: Standardized material (2 to 10 g)  -Ascorbic Acid: 1 g  -Ethanol: 150 mL  -Potassium Hydroxide (KOH): 50 mL of 60% solution  -Water (H₂O): 150 mL (for extraction)  -Hexane: 100 mL (for three extractions)  -Anhydrous Sodium Sulfate (Na₂SO₄): for drying the organic phase  -Mobile Phase: 3% Dioxane in Hexane  -HPLC Column: Lichosorb Si 60  -Detection Wavelength: 292 nm  -Flow Rate: 1.0 mL/min  -Injection Volume: 20 µL | **Sample Preparation:**  Accurately weigh the material to be examined (2 g and 10 g) using an analytical balance mass in grams.  **Saponification Reaction:**  Combine the following in an Erlenmeyer Flask:  -Ascorbic Acid: 1 g  - Ethanol: 150 mL  - KOH Solution: 50 mL 60% KOH (0.06 g/mL; thus, 50 mL → 3 grams of KOH or is equivalent to now a total molarity in this solution accounting for the percent weight will be equal to.0535 mol, MW =56.11 g/mol)  Now the solution has a volume of about 200 mL. After adding alkaline solutions mentioned above, turn the strong light and stir the mixture at room temperature for 12 hours (dark helps saponification) with aluminum foil covered.  **Extraction Process:**  -After the reaction, transfer the mixture to a separatory funnel.  -Add 150 mL of H₂O (0.15 L) to the funnel and shake gently to mix the phases, allowing for separation.  -Perform three sequential extractions with 100 mL of hexane each time:  -After each addition of hexane, shake the funnel and allow the layers to separate.  -Collect the hexane layer each time, discarding the aqueous layer.  **Washing the Organic Phase**:  -Combine the collected hexane extracts and wash them three times with 100 mL of distilled H₂O (0.1 L) to remove any residual polar impurities. After each wash, separate and discard the aqueous layer.  Drying the Organic Phase:  -Pass the combined hexane solution through a drying agent, such as anhydrous Na₂SO₄, to remove any remaining water. Collect the dried organic solution.  Evaporation:  Evaporate the hexane using a rotary evaporator under reduced pressure until a residue remains (in g).  **Preparation of the Mobile Phase:**  Prepare a mobile phase of 3% dioxane in hexane:  For example, for a total of 100 mL of mobile phase, mix 3 mL of dioxane with 97 mL of hexane | **HPLC Analysis:**  -Dissolve the residue from the evaporation step in the prepared mobile phase and filter through a 0.45 µm filter to remove particulates.  -Inject 20 µL of the solution into the HPLC system.  -Set up the HPLC with a Lichosorb Si 60 column, using UV detection at 292 nm and a flow rate of 1.0 mL/min.  Calibration and Quantification:  -Generate a calibration curve using known concentrations of the target analyte. Ensure that the concentrations cover the expected range based on the initial sample mass.  -Plot the peak area versus concentration and determine the linearity of the curve.  -Calculate the sample concentration from the peak area of the sample, using the equation derived from the calibration curve.  **Data Analysis:**  -Compare the retention time of the sample peaks with the standards to confirm the identity of the compounds.  Units:  Mass is measured in grams (g).  Volume is measured in milliliters (mL).  Concentration may be reported in grams per liter (g/L) or milligrams per milliliter (mg/mL). |
|  |  |  |  |
|  |  |  |  |
| Total Fibers *(Enzymatic-Gravimetric method)* | **Methods for Measuring Total Dietary Fiber Content**  **Reagents and Buffers Preparation**  - MES-TRIS Buffer (50 mM, pH 6.3)  **Materials:**  MES (2-(N-morpholino) ethanesulfonic acid): 9.76 g  TRIS (tris(hydroxymethyl)aminomethane): 6.055 g  Deionized water: Up to 1 L  **Procedure:**  **-**Weigh 9.76 g of MES and dissolve it in approximately 800 mL of deionized water.  -In a separate container, weigh 6.055 g of TRIS and dissolve it in approximately 200 mL of deionized water.  -Combine the MES and TRIS solutions in a 1 L volumetric flask.  -Adjust the final volume to 1 L with deionized water.  -Use a calibrated pH meter to adjust the pH to 6.3, if necessary. | **Stabilizing Buffer:**  **Materials:**  MES: 9.76 g (same as above)  TRIS: 6.055 g (same as above)  NaCl (sodium chloride): 0.585 g (for 10 mM in 1 L)  CaCl₂ (calcium chloride): 0.221 g (for 3 mM in 1 L)  Deionized water: Up to 1 L  Procedure:  -Prepare the MES-TRIS buffer as described in section  -Weigh and add 0.585 g of NaCl and 0.221 g of CaCl₂ to the combined buffer solution.  -Mix thoroughly until completely dissolved.  -Adjust the final volume to 1 L with deionized water.  Check and ensure the pH is maintained at 6.3 at 24°C. | **Enzymatic Digestion**  Enzymes Used:  Protease (Megazyme E-BSPRT)  Amyloglucosidase (Megazyme E-AMGDF)  Thermostable Amylase (Megazyme E-BLAAM)  Procedure:  -Prepare a reaction mixture containing 1 g of the dietary sample.  -Add 10 mL of the MES-TRIS buffer prepared in section 1.1.  -Add 0.1 mL of thermostable amylase and incubate at the appropriate temperature (usually 95°C) for 30 minutes to hydrolyze starch.  -Cool the mixture to room temperature and add 0.1 mL of protease. Incubate at 60°C for 30 minutes.  -Add 0.1 mL of amyloglucosidase and incubate at 60°C for an additional 30 minutes to complete the hydrolysis of remaining starch and soluble fibers.  **Final Analysis**  -After enzymatic digestion, the resulting mixture is filtered, and the soluble fraction is collected.  -The total dietary fiber content is determined by the gravimetric method, where the residue is dried and weighed.  **Units and Calculations**  Concentrations: All buffer solutions are expressed in millimolar (mM).  Weights: All solid reagents are measured in grams (g).  Volume: Liquid reagents and buffers are measured in liters (L) or milliliters (mL)  Results Reporting: Total dietary fiber content should be reported in grams per 100 grams of the original sample (g/100 |
|  |  |  |  |
|  |  |  |  |
|  |  |  |  |
